# Preoperative Factors Associated with Weight Loss and Recurrent Weight Gain after Bariatric Surgery: A Longitudinal Study in a UK Public Healthcare Setting

**DOI:** 10.1007/s11695-026-08900-1

**Published:** 2026-08-28

**Authors:** Luqman Naim Bin Aizan, Akheel A. Syed, Bilal Alkhaffaf

**Affiliations:** 1 Department of Oesophago-Gastric and Bariatric Surgery, Salford Care Organisation, Northern Care Alliance NHS Foundation Trust, Greater Manchester, United Kingdom; 2 Department of Diabetes, Endocrinology and Obesity Medicine, Salford Care Organisation, Northern Care Alliance NHS Foundation Trust, Greater Manchester, United Kingdom; 3 Faculty of Biology, Medicine and Health, University of Manchester, Manchester, United Kingdom

## Abstract

**Objective:**

Bariatric surgery is an effective treatment for obesity. This study aims to identify demographic factors of weight loss and recurrent weight gain over a 10-year follow-up period.

**Methods:**

This retrospective observational study includes 2092 patients for overall trends, 1032 patients for predictors of weight loss, and 605 patients for predictors of recurrent weight gain. Percentage total weight loss (%TWL) trends were measured over 10 years. The rate of weight loss and recurrent weight gain was used as a response variable in multivariate analysis.

**Results:**

After bariatric surgery, mean ± SD %TWL was greatest at 31.3 ± 10.7 % by 2 years, followed by gradual recurrent weight gain to a %TWL of 25.1 ± 12.6 % at 10 years. Up to 5 years, %TWL was greater with Roux-en-Y gastric bypass (n=1080; 27.9 ± 11.5 %) and one-anastomosis gastric bypass (n=217; 28.3 ± 10.1) had greater %TWL than sleeve gastrectomy (n=795; 22.8 ± 12.5 %) (ANOVA, p < 0.005). Age, sex, preoperative body mass index, diabetes, operation type, and smoking were statistically significant predictors of weight loss up to 2 years (p < 0.05). Age, hypertension, Index of Multiple Deprivation quintile, and early %TWL up to 2 years were statistically significant predictors of recurrent weight gain after 2 years (p < 0.05).

**Conclusions:**

Operation type, age, sex, preoperative body mass index, diabetes, operation type and smoking had significant associations with weight loss while age, hypertension, Index of Multiple Deprivation and initial postoperative weight loss had significant associations with recurrent weight gain.

## Key Points

• OAGB may show comparable outcomes to RYGB and SG.

• Age, sex, preoperative body mass index, diabetes, operation type, and smoking were statistically significant factors associated with weight loss up to 2 years.

• Age, hypertension, Index of Multiple Deprivation quintile, and early %TWL up to 2 years were statistically significant factors associated with recurrent weight gain between 2 and 10 years.

## Introduction

Bariatric surgery is an established treatment for obesity resulting in long-term weight loss and remission of comorbid diseases [1–3]. However, their outcomes may be affected by certain factors including operation type. The one-anastomosis gastric bypass (OAGB) is a viable surgical option [4], though some surgeons continue to have reservations about OAGB due to limited long-term follow-up data [5], despite some studies showing the OAGB to have comparable weight loss outcomes to Roux-en-Y gastric bypass (RYGB) and similar complication rates to both sleeve gastrectomy (SG) and RYGB [6, 7]. Previous studies have already shown that factors such age, sex, preoperative weight, ethnicity, socioeconomic status, diabetes and smoking could affect weight loss outcomes [8–24], but few have included the OAGB. Additionally, recurrent weight gain plays an important role on long-term outcomes after bariatric surgery. The association of postoperative factors such as diet, physical activity and mental health on weight relapse has been investigated previously [25–27], but studies investigating preoperative factors on recurrent weight gain over a 10-year follow-up period are scarce [28, 29], especially in the UK healthcare setting. In 2023, the SOPHIA study detailed the development of a multinational externally validated machine learning model to predict weight loss outcomes after bariatric surgery using seven preoperative variables but is limited due to lack of OAGB surgery and only predicted up to 5 years after operation [30].

We investigated trends in weight loss and weight relapse over a 10-year follow-up period in patients after SG, RYGB or OAGB and the predictors that influence these trends with the aim of guiding future predictive studies.

## Methods

Our study adhered to the Strengthening the Reporting of Observational studies in Epidemiology (STROBE) guidelines [31].

### Data Source and Feature Selection

This was a retrospective cohort study conducted at a specialised severe and complex obesity and bariatric surgery centre in Northwest England from 2008 to 2024, with a follow-up period of up to 120 months after bariatric surgery. Patients aged 18 years and over who underwent laparoscopic SG, RYGB or OAGB were included. All laparoscopic-to-open conversion and revision bariatric operations were excluded. A final cohort of 2,092 patients with at least 1 follow-up weight measurement was included for the analysis of average weight loss at different points in time postoperative. For analysis of rate of weight loss up to 2 years, we included 1,032 patients who had at least 4 weight measurements at baseline, 6 months, 1 year, and 2 years. From this cohort subset, patients with at least one additional measurement at 5 or 10 years were further included in the analysis investigating rate of weight relapse from 2 to 10 years, with a sample size of 605. Demographic data collected included age, sex, ethnicity, Index of Multiple Deprivation (IMD) decile, history of hypertension, diabetes, and anxiety or depression, and smoking status based on previously identified factors likely to influence weight loss outcomes [32]. Ethnicity was captured using administrative self-reported data and did not have approval to ask or explore family links as this was out of scope and not routinely collected data. The Index of Multiple Deprivation (IMD) is the official measure of relative deprivation in England which defines deprivation to encompass a wide range of living conditions and not just financial resources. All neighbourhoods are ranked according to their level of deprivation, so the IMD is measure of deprivation on a relative scale, helpful in planning, policy and research. A lower decile indicates greater level of deprivation [33].

Data were extracted from electronic patient records. Weight measurements were recorded at baseline (preoperative), 6 months, 1 year, 2 years, 5 years, and 10 years after bariatric surgery. Any available weight measurements were included. Baseline preoperative weight was the weight measured on the day of surgery or the closest available measurement before surgery. For weight measurements at 6 months and 1 year, any measurement within 2 months of the target time points were included. For the 2-year time point, measurements within 4 months were included, and for the 5- and 10-year time points, measurements within 12 months were included to avoid excessively high rates of attrition. Patient sex, ethnicity, IMD (derived from patient’s postcode) and comorbid diseases were extracted directly from patient records. Smoking history included current and any previous history of smoking. All data were anonymized and stored in a secure electronic database.

## Preoperative Assessment and Selection Process

All patients were assessed within a multidisciplinary bariatric service. Eligibility for surgery was based on national and institutional criteria, including body mass index (BMI ≥ 40 kg/m² or ≥ 35 kg/m² with obesity-related comorbidities), prior attempts at non-surgical weight loss, and fitness for general anaesthesia, in accordance with guidance from the National Institute for Health and Care Excellence and British Obesity and Metabolic Surgery Society [34, 35].

Patients underwent a comprehensive preoperative evaluation including medical, nutritional and psychological assessment. Comorbidities such as type 2 diabetes, hypertension, obstructive sleep apnoea and gastro-oesophageal reflux disease were documented and optimised prior to surgery. Nutritional assessment included screening for micronutrient deficiencies and counselling regarding postoperative dietary requirements. Psychological assessment evaluated eating behaviours, mental health conditions, and readiness for surgery, consistent with British Obesity and Metabolic Surgery Society recommendations.

Procedure selection (sleeve gastrectomy [SG], Roux-en-Y gastric bypass [RYGB], or one-anastomosis gastric bypass [OAGB]) was individualised following multidisciplinary discussion. Factors influencing procedure choice included BMI, presence of type 2 diabetes, severity of reflux symptoms, eating behaviours, patient preference, and perceived risk of malabsorption. In general, RYGB and OAGB were favoured in patients with significant metabolic disease, while SG was more commonly selected in patients with lower operative risk or where malabsorption was less desirable. All patients completed a preoperative liver reduction diet for approximately 2–3 weeks prior to surgery.

## Surgical Techniques

All procedures were performed laparoscopically by experienced bariatric surgeons using a standardised technique.

Sleeve gastrectomy (SG): A standard laparoscopic sleeve gastrectomy was performed using a 4–5 port technique. The greater curvature was mobilised starting approximately 4–6 cm proximal to the pylorus and continuing to the angle of His. A bougie (typically 36–40 Fr) was used to calibrate the sleeve. Gastric division was performed using a linear stapler to create a tubular gastric conduit. Staple line reinforcement and leak testing were performed according to surgeon preference.

Roux-en-Y gastric bypass (RYGB): A small gastric pouch (~ 20–30 mL) was created using a linear stapler and a combined bypass length of 200 cm was created. The jejunum was divided approximately 100–150 cm distal to the ligament of Treitz to form the biliopancreatic limb. An alimentary (Roux) limb of approximately 50–100 cm was constructed. A gastrojejunostomy was created (either stapled or hand-sewn), followed by a jejunojejunostomy to restore bowel continuity. Mesenteric defects were routinely closed to reduce the risk of internal herniation.

One-anastomosis gastric bypass (OAGB): A long gastric pouch was fashioned along the lesser curvature. A loop gastrojejunostomy was created with the jejunum approximately 150–200 cm distal to the ligament of Treitz, depending on patient BMI and metabolic profile. This created a single anastomosis with a biliopancreatic limb of corresponding length. Care was taken to ensure appropriate pouch configuration and tension-free anastomosis.

## Postoperative Care and Follow-Up Programme

Postoperative care followed a standardised enhanced recovery pathway. Patients were mobilised early and commenced on a staged diet beginning with liquids and progressing to pureed and solid foods over several weeks under dietetic supervision.

All patients were prescribed routine vitamin and mineral supplementation, including multivitamins, iron, calcium with vitamin D, and vitamin B12, with additional supplementation as required based on biochemical monitoring, in line with British Obesity and Metabolic Surgery Society guidance [36].

Patients were followed up in a structured multidisciplinary programme consistent with recommendations from National Institute for Health and Care Excellence and British Obesity and Metabolic Surgery Society, with scheduled reviews at approximately 6 months, 1 year, 2 years, 5 years and 10 years postoperatively [34, 35]. Follow-up included weight measurement, assessment of comorbidities, nutritional monitoring, and dietary and behavioural support.

Patients demonstrating suboptimal weight loss or weight relapse were offered additional interventions including intensified dietary support, psychological input, and where appropriate, consideration of pharmacological therapy or revisional surgery.

### Statistical Analysis

Outcome measures were percentage total weight loss (%TWL), average rate of percentage total weight loss (rate of %TWL) up to 2 years and average rate of percentage total weight relapse (rate of %TWR) between 2 and 10 years. %TWL and %TWR were defined as (weight change/preoperative weight × 100). For the analysis of rate of %TWL (cohort of 1032), we plotted a linear model for each individual patient which produced a line of best fit with a gradient that signifies the percentage weight loss per month (rate of %TWL) for that patient. This gradient was extracted and used as a response variable in both univariate and multivariate analysis of rate or %TWL. A similar method was used for the 605 patients included in the weight relapse analysis, but the linear model was plotted between 2 and 10 years instead for analysis of rate of %TWR.

The analysis was divided into two parts. The first part analyses overall weight loss trends and compares %TWL over a 10-year period between the three types of operations. We compared the average %TWL at each time point and analyzed the rate %TWL between 0 and 6 months, rate of %TWL between 0 and 2 years and the rate %TWR between 2 and 10 years, using ANOVA testing for significance between operation types. The second part involved multivariate regression models to identify statistically significant predictors for both weight loss and weight relapse. Estimated effects for age were expressed in 5-year increments, and preoperative BMI was expressed in 5-point increments. All relevant variables were included in the initial multivariate model followed by backwards elimination of statistically non-significant variables until only statistically significant predictor variables remained.

Additional analysis of percentage total weight relapse from nadir weight loss by the three operation types was conducted. Three cutoffs were used: 10%, 25% and 50% weight relapse from nadir, measured at 5 and 10 years. The proportion of patients achieving each cutoff was calculated for each operation type and analysed using Chi-squared test and Fisher’s exact test.

All statistical analyses were conducted using R software version 4.2.2 for Mac OS. A p-value of < 0.05 was considered statistically significant. Estimates were presented with 95% confidence intervals.

## Results

### Baseline Characteristics

The entire cohort (*n* = 2092) had a mean ± standard deviation (SD) age of 46.2 ± 10.8 years and was comprised of 75.8% women (Table 1). RYGB (51.6%) made up majority of operations, followed by SG (38%) and OAGB (10.4%). The cohort was predominantly of White ethnicity (94.4%).

**Table 1:** Cohort demographics and baseline characteristics

Age at operation, mean (SD)	Overall cohort, *N* = 2092	Rate of weight loss analysis, *N* = 1032	Rate of weight relapse analysis, *N* = 605
	46.2 (10.8)	47 (10.9)	47.8 (10.8)
**Preoperative weight and BMI**			
Baseline weight in kg, mean (SD)	137 (26.2)	137 (25.9)	138 (26.2)
Body mass index in kg/m^2^, mean (SD)	49.1 (7.4)	49.4 (7.4)	49.9 (7.4)
**Sex**			
Female, n (%)	1586 (75.8)	777 (75.3)	457 (75.5)
Male, n (%)	506 (24.2)	255 (24.7)	148 (24.5)
**Operation**			
Sleeve gastrectomy, n (%)	795 (38.0)	347 (33.6)	201 (33.2)
Roux-en-Y gastric bypass, n (%)	1080 (51.6)	582 (56.4)	346 (57.2)
One anastomosis gastric bypass, n (%)	217 (10.4)	103 (10.0)	58 (9.6)
**Ethnicity**			
White, n (%)	1975 (94.4)	974 (94.4)	573 (94.7)
Other, n (%)	117 (5.6)	58 (5.6)	32 (5.3)
**IMD quintile**			
Quintile 1, n (%)	992 (47.5)	480 (46.6)	273 (45.3)
Quintile 2, n (%)	470 (22.5)	233 (22.6)	150 (24.9)
Quintile 3, n (%)	281 (13.5)	140 (13.6)	80 (13.3)
Quintile 4, n (%)	220 (10.5)	123 (11.9)	69 (11.4)
Quintile 5, n (%)	126 (6.0)	54 (5.2)	31 (5.1)
NA, n (%)	3 (0.1)	2 (0.1)	2 (0.3)
**Comorbid diseases**			
Diabetes, n (%)	784 (37.5)	394 (38.2)	225 (37.2)
Hypertension, n (%)	930 (44.5)	502 (48.6)	306 (50.6)
Smoking history, n (%)	894 (42.7)	464 (45.0)	276 (45.6)
Depression or anxiety, n (%)	681 (32.6)	320 (31.0)	182 (30.1)
**Follow-up rates**			
Due future measurement at 6 months, n (% of total cohort)	35 (1.7)	0 (0)	0 (0)
Lost to follow-up at 6 months, n (%)	287 (14.0)	0 (0)	0 (0)
Due future measurement at 12 months, n (% of total cohort)	104 (5.0)	0 (0)	0 (0)
Lost to follow-up at 12 months, n (%)	384 (19.3)	0 (0)	0 (0)
Due future measurement at 24 months, n (% of total cohort)	254 (12.1)	0 (0)	0 (0)
Lost to follow-up at 24 months, n (%)	646 (35.1)	0 (0)	0 (0)
Due future measurement at 60 months, n (% of total cohort)	450 (21.5)	122 (11.8)	0 (0)
Lost to follow-up at 60 months, n (%)	877 (53.4)	325 (35.7)	20 (3.3)
Due future measurement at 120 months, n (% of total cohort)	1230 (58.8)	527 (51.1)	269 (44.5)
Lost to follow-up at 120 months, n (%)	616 (71.5)	316 (62.6)	147 (43.8)

## Weight Loss Trends

**Fig. 1:**
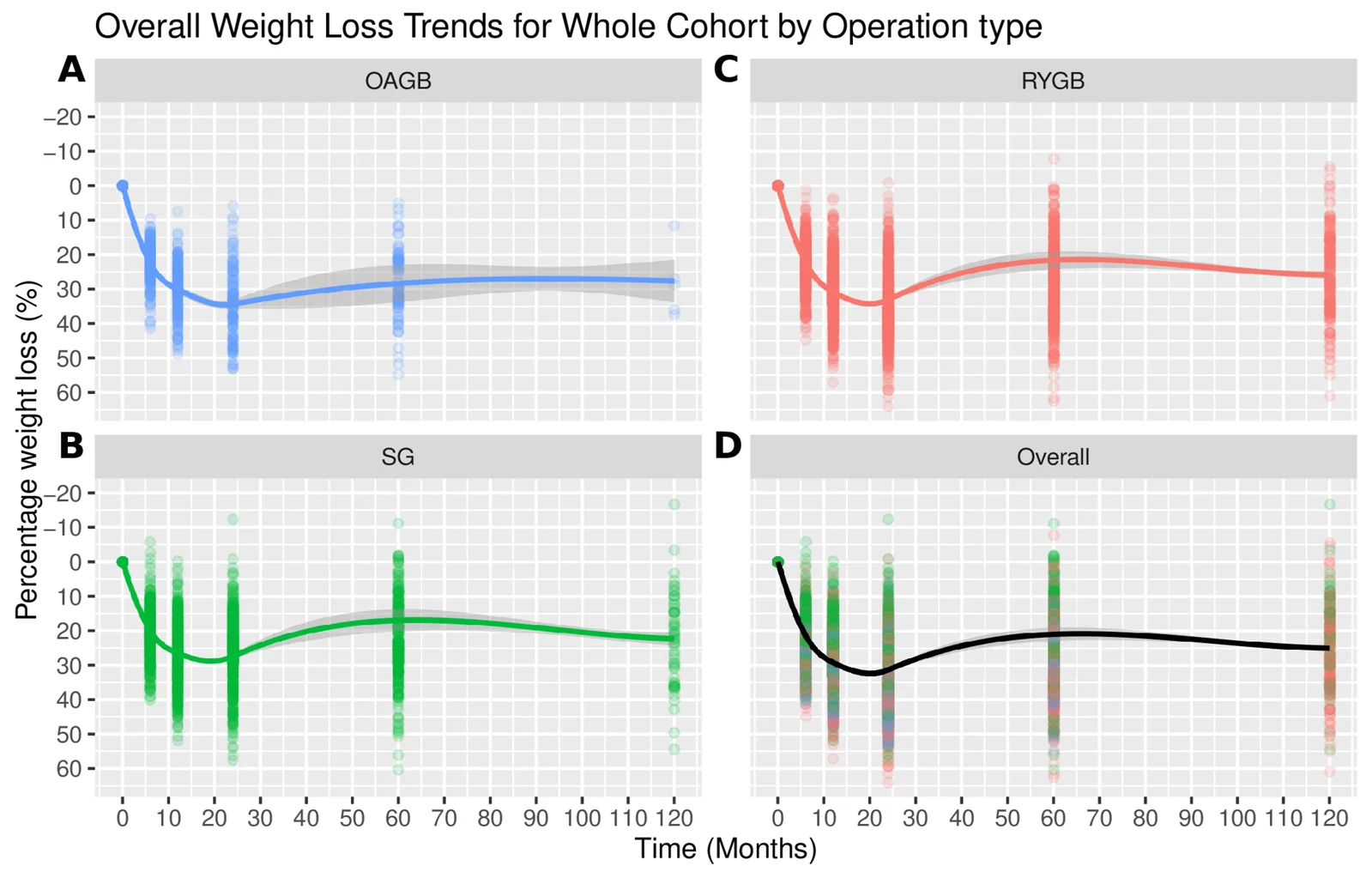
Weight loss trends for (**A**): OAGB, (**B**): SG, (**C**): RYGB, and (**D**): entire cohort. This figure was generated using the smoothing method “loess” in R which visualizes the general trends over time but may deviate from manually calculated means and confidence intervals due to its adjustments for local data variations

Weight loss was highest at 2 years before gradual weight relapse up to 10 years (Fig. 1). RYGB and OAGB resulted in significantly higher %TWL compared to SG up to the 5 years (*p* < 0.005).

**Fig. 2:**
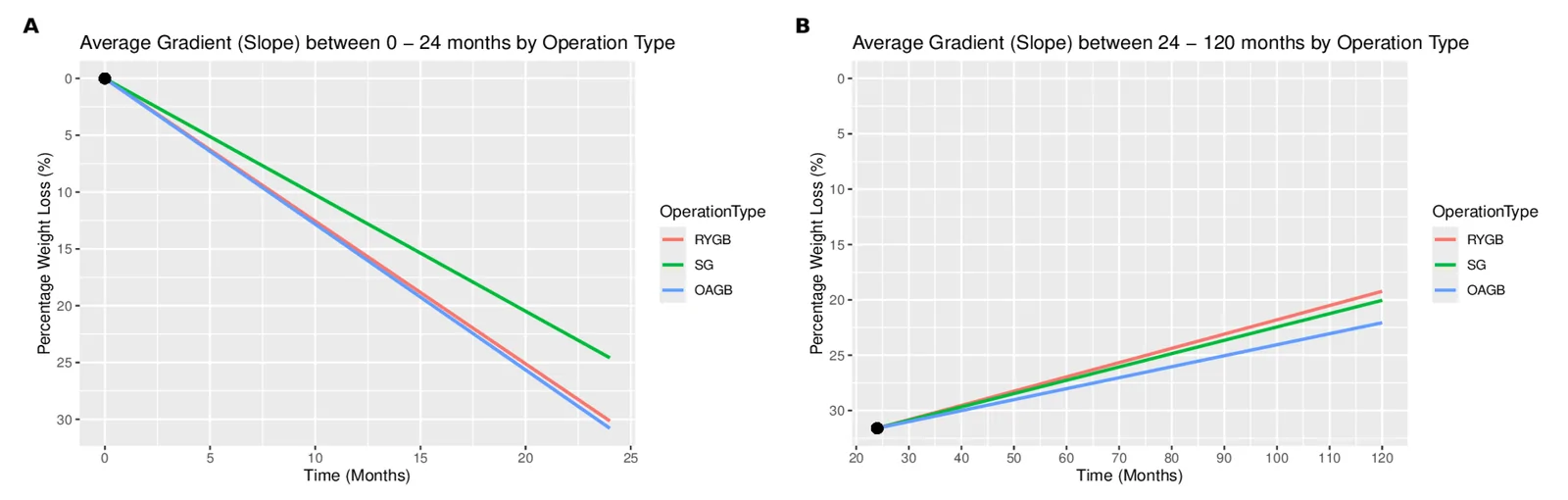
Panel (**A**): Rate of %TWL. Panel (**B**): Rate of %TWR

The average rate of %TWL and %TWR were similar for RYGB and OAGB (Table 2). ANOVA with post-hoc Tukey demonstrates a higher rate of %TWL up to 2 years in patients who underwent RYGB and OAGB compared to SG (*p* < 0.005) with no significant difference in rate of %TWR (Fig. 2).

**Table 2:** Mean percentage weight loss (%) stratified by time point and mean rate of percentage of weight loss per month (%/month)

Mean percentage weight loss stratified by time	Overall *N* = 2092 Mean ± SD (95% CI)	SG *N* = 795 Mean ± SD (95% CI)	RYGB *N* = 1080 Mean ± SD (95% CI)	OAGB *N* = 217 Mean ± SD (95% CI)	*P* value (ANOVA)
6 months	21.5 ± 6.2 (21.2–21.7)	19.9 ± 6.3 (19.4–20.4)	22.4 ± 6.0 (22.0–22.7)	22.5 ± 6.0 (21.6–23.4)	< 0.005
12 months	29.2 ± 8.6 (28.8–29.7)	26.5 ± 8.7 (25.8–27.2)	30.9 ± 8.2 (30.3–31.4)	30.1 ± 7.8 (28.9–31.3)	< 0.005
24 months	31.3 ± 10.7 (30.7–31.9)	27.4 ± 10.6 (26.3–28.4)	33.0 ± 10.2 (32.3–33.8)	34.4 ± 10.6 (32.5–36.3)	< 0.005
60 months	26.3 ± 11.9 (25.4–27.1)	22.8 ± 12.5 (21.3–24.4)	27.9 ± 11.5 (26.8–29)	28.3 ± 10.1 (26.1–30.5)	< 0.005
120 months	25.1 ± 12.6 (23.5–26.7)	22.3 ± 13.2 (18.9–25.8)	25.9 ± 12.3 (24.1–27.7)	28.1 ± 10.3 (15.4–40.8)	0.146
**%TWL/mth**	*N* = 1032	*N* = 347	*N* = 582	*N* = 103	
0–6 months	3.69 ± 1.00 (3.62–3.75)	3.47 ± 1.04 (3.36–3.58)	3.79 ± 0.95 (3.71–3.86)	3.86 ± 1.03 (3.66–4.06)	< 0.005
0–24 months	1.18 ± 0.44 (1.15–1.21)	1.02 ± 0.44 (0.98–1.07)	1.26 ± 0.42 (1.22–1.29)	1.28 ± 0.42 (1.20–1.37)	< 0.005
**%TWR/mth**	*N* = 605	*N* = 201	*N* = 346	*N* = 58	
24–120 months	−0.12 ± 0.22 [(−0.14) – (−0.11)]	−0.12 ± 0.25 [(−0.16) – (−0.09)]	−0.13 ± 0.19 [(−0.15) – (−0.11)]	−0.10 ± 0.23 [(−0.16) – (−0.04)	0.611

### Predictors of Weight Loss Up to 2 Years

**Fig. 3:**
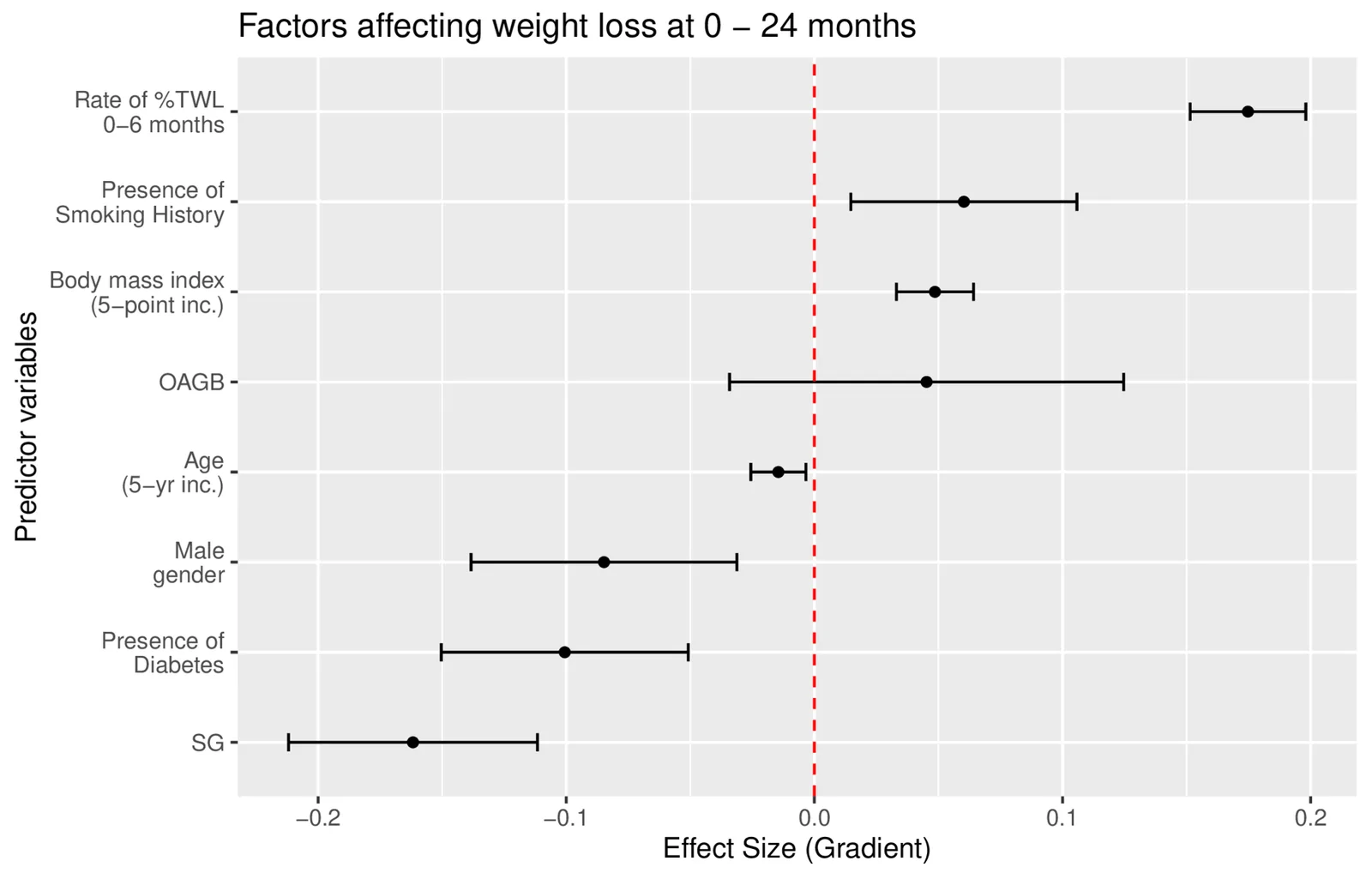
Factors associated with changes in percentage weight loss between 0–2 years

Factors associated with decreased rate of %TWL include older age (per 5-year increment), male sex and presence of diabetes while factors associated with increased rate of %TWL include higher preoperative BMI (per 5-point increment), higher initial rate of %TWL in the first 6 months and smoking history (Fig. 3).

### Predictors of Weight Relapse After 2 Years

**Fig. 4:**
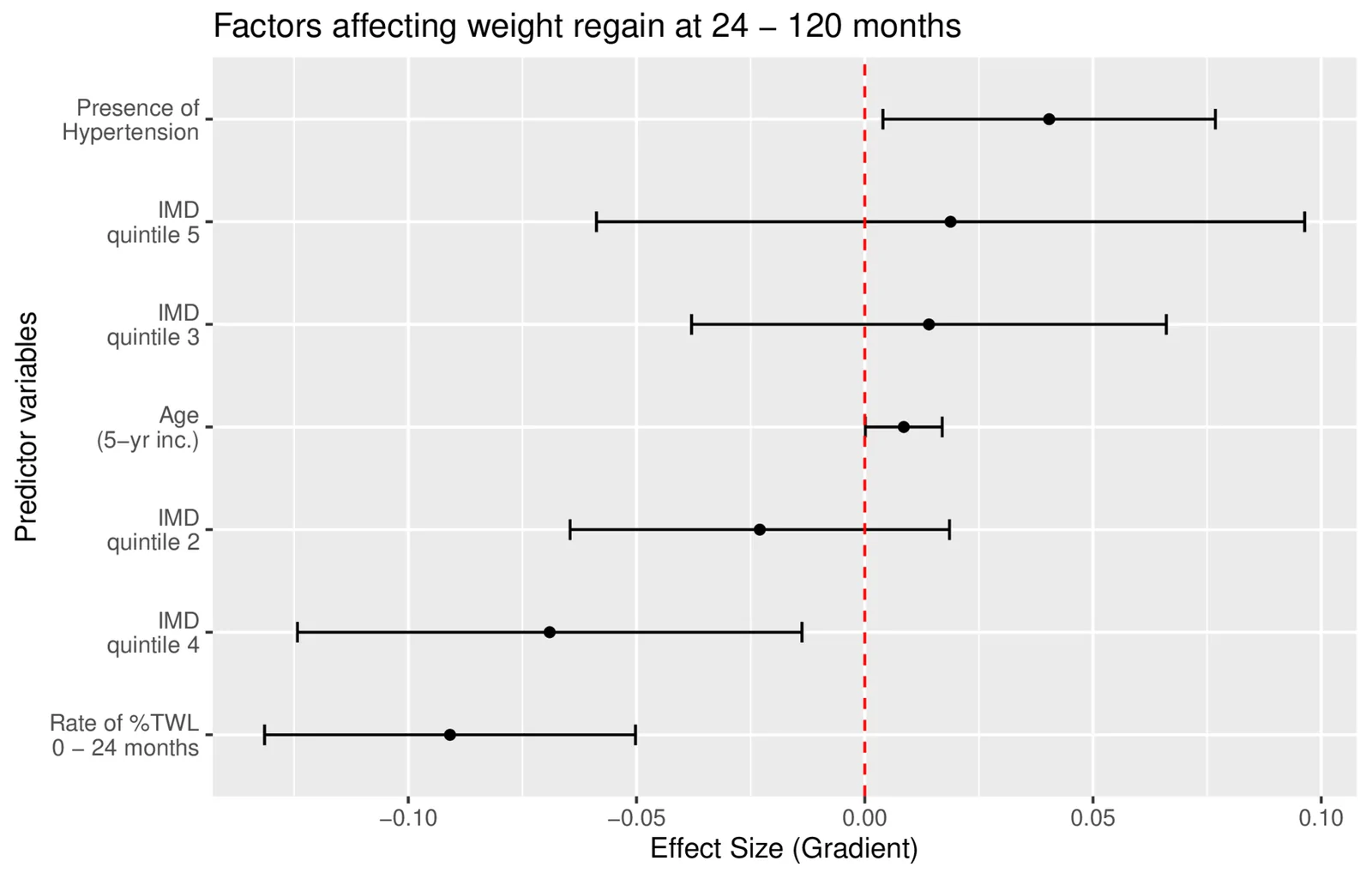
Factors associated with changes in percentage weight relapse between 2–10 years

Factors associated with lower rate of %TWR were increasing age (per 5-year increment) and hypertension while factors associated with higher rate of %TWR were being in the fourth IMD quintile and a higher rate of %TWL in the first 24 months (Fig. 4).

### Weight Relapse Percentages

At 5 years, OAGB had the least proportion of patients regaining 10% and 50% weight from nadir (Table 3). Similarly, at 10 years, OAGB had the least proportion of patients regaining 10%, 25% and 50% weight from nadir. At 5 years, a higher proportion of female patients regained 10%, 25% and 50% weight from nadir. At 10 years, a higher proportion of male patients regained 25% weight from nadir. At 5 years, lower IMD decile (higher deprivation) had higher proportion of patients regaining 10% weight from nadir. At 10 years, lower IMD decile (higher deprivation) had higher proportion of patients regaining 10% and 50% weight from nadir.

**Table 3:** Proportion of patients that regained 10%, 25% and 50% of their weight from nadir at 5 years and 10 years after bariatric surgery

Proportion of patients with weights at 5 years	10% weight regain	25% weight regain	50% weight regain
Overall (%) *N* = 737	45.5	11.5	0.7
RYGB (%) *N* = 417	46.5	12.9	0.5
SG (%) *N* = 241	46.1	9.5	1.2
OAGB (%) *N* = 79	38.0	10.1	0.0
p-value	0.37	0.39	0.51
Female (%) *N* = 557	46.9	12.7	0.7
Male (%) *N* = 180	41.1	7.8	0.6
p-value	0.21	0.09	0.77
IMD 1–5 (%) *N* = 565	46.0	11.3	0.5
IMD 6–10 (%) *N* = 170	42.9	12.4	1.2
p-value	0.54	0.82	0.33
Proportion of patients with weights at 10 years			
Overall (%) *N* = 238	48.3	19.3	5.0
RYGB (%) *N* = 176	49.4	19.9	6.3
SG (%) *N* = 57	47.4	19.3	1.8
OAGB (%) *N* = 5	20.0	0.0	0.0
p-value	0.49	0.82	0.46
Female (%) *N* = 171	50.3	18.7	5.3
Male (%) *N* = 67	43.3	20.9	4.5
p-value	0.69	0.72	1.00
IMD 1–5 (%) *N* = 190	51.6	18.9	5.3
IMD 6–10 (%) *N* = 48	35.4	20.8	4.2
p-value	0.07	0.84	1.00

### Follow Up Rates

Our follow up rates throughout the study period were 86% at 6 months, 81% at 1 year, 65% at 2 years, 47% at 5 years, and 29% at 10 years.

## Discussion

This study is the first to investigate the association between operation type, including OAGB and several preoperative factors and both weight loss and recurrent weight gain trends over a 10-year follow-up period after bariatric surgery in a public healthcare system in the UK. Previous studies were limited by lack of OAGB operation, short follow-up durations, using weight loss at individual timepoints, conducting studies in private healthcare systems and exclusion of a weight relapse analysis [8, 9, 11–13, 15, 16, 18, 20, 22–24, 37, 38]. Very limited studies have looked at long term weight loss outcomes up to 10 years postoperative [39, 40], while only one large scale study classified and investigated weight relapse after bariatric surgery [41]. As mentioned previously, the SOPHIA study is the best attempt at a predictive model for weight loss outcomes after bariatric surgery, reporting good accuracy especially for gastric bypass but only up to 5 years postoperatively and does not include the OAGB [30, 42]. Our study attempts to investigate the association of OAGB and other preoperative factors on weight loss and weight relapse over a 10-year period. In our study, RYGB and OAGB showed greater weight loss up to 5 years after bariatric surgery compared to SG. Large trials like the SLEEVEPASS trial show that RYGB results in greater weight loss compared to SG [2], while another systematic review which focused on weight loss outcomes up to 10 years after RYGB and SG also showed greater weight loss in RYGB [39]. Studies on OAGB show comparable weight loss outcomes with RYGB, with shorter operating times, lower revision rates and lower complication rates over follow-up periods of up to 5 years [6, 7, 43–45]. There have been limited randomised trials comparing RYGB with OAGB [46]. The largest and most recent of which was the YOMEGA trial with 250 randomised patients that reported outcomes at 2 and 5 years postoperatively showing non-inferiority of OAGB compared to RYGB in terms of weight loss, remission of disease and postoperative complications at 5 years [47, 48]. Although our study demonstrates comparable weight loss outcomes with OAGB compared to SG with no significant difference to RYGB over the short-term with lower weight relapse compared to non-OAGB, our small sample size at 10-year follow-up limits the strength of this finding over the long-term. Procedures performed were also not randomised as operative selection was likely influenced by factors such as BMI, patient preference, diabetes and perceived malabsorption, introducing confounding by indication. In addition, we could not compare other measures such as side effects, complication rates and effects on co-morbidity and quality of life between operation types which should be addressed in further studies.

Most studies reported greater weight loss for female patients[11, 22, 24, 49, 50] some showed no association [12, 18, 21, 51], while a minority found greater weight loss in male patients [23, 52]. While these differences in sex-related outcomes could be explained by variation in fat metabolism, hormonal influences and behavioural factors between sexes [53, 54], one study proposed that sex-related outcomes are reliant on the operation performed [24]. On multivariate analysis in our study, female patients achieved significantly higher weight loss than male patients up to 2 years with no significant difference in weight relapse between 2 and 10 years but was limited by a large disparity in sex with the female cohort far outnumbering the male cohort. This could likely explain the difference in outcomes paired with missing data on non-follow up. When matched for key factors, one study found no significant difference in weight loss outcomes between male and female patients leaving no clear evidence that bariatric surgery benefits one sex more than the other [55]. Similar results showing no difference in weight loss between men and women were found in the recent SOPHIA study [30]. There is a large disparity in uptake of bariatric surgery between male and female patients with male patients only making up 24% of all patients receiving bariatric surgery in England despite roughly similar rates of obesity in both sexes [56]. More equal cohorts will be needed in future studies for better comparisons and to address this disparity in uptake.

In our study, diabetes was a significant predictor of lower rates of %TWL up to 2 years. Diabetes is an established predictor of poor weight loss outcomes [8, 11, 21, 22, 24, 57], with some studies suggesting that the impact of diabetes varies with disease severity. Studies show that patients with diabetes with higher HbA1c levels (> 53mmol/mol) or those receiving insulin experienced poorer weight loss outcomes [18, 58], while successful postoperative diabetes remission was associated with better weight loss outcomes [57]. Disruption to insulin and endocrine signalling pathways could explain differences in weight loss outcomes [24, 59, 60]. Our study lacked several measures including HbA1c, remission success and insulin dependence in the perioperative period, reducing the granularity of the effect of weight loss on diabetes outcomes (and vice versa). The SOPHIA study also demonstrated that the duration a patient suffered diabetes had a strong predictive value on weight loss with longer duration associated with less weight loss and should be recorded in future studies [30].

Results from our study showed that patients with a higher preoperative BMI experienced greater rate of %TWL up to 2 years. Most studies showed a negative correlation between increased preoperative weight and BMI on weight loss outcomes [8, 12, 15, 20, 21, 23, 49, 61]. A smaller number of studies, like ours, showed a positive correlation [10, 24]. However, the use of BMI can lead to misleading results and should be interpreted with caution with studies recommending %TWL as the best alternative [62]. Additionally, increased prevalence of obesity-related comorbidities in patients with higher BMIs further reduce its sole predictive value in weight loss outcomes [63]. A standardised method of reporting weight changes is needed to make accurate comparisons in future studies.

Older age is associated with worse weight loss outcomes [10, 12, 20, 22, 24, 50]. Our study reveals an association between older age and a small reduction in rate of %TWL up to 2 years. Variations in metabolic capacity, physical ability, motivation, and comorbidity prevalence may explain these findings [64, 65]. Although studies including the SOPHIA study show that younger patients benefit from greater BMI loss and better remission rates of comorbidities [30, 65], bariatric surgery remains a safe and effective option for patients of older age and exclusion should not be based on age alone but may require tailored postoperative follow-up [66–68].

Weight relapse usually begins around two years after bariatric surgery [69, 70]. Our study showed mean weight relapse from total preoperative weight of 6.2% between 2 and 10 years. At 5 years, 57.7% of patients experienced weight relapse of 10% from nadir, increasing to 72.6% at 10 years. Age, hypertension, IMD decile and rate of %TWL up to 2 years were significant predictors of weight relapse. One discrepancy we found was an association between increased rate of %TWR with IMD quintile 4 but not with the least deprived quintile (IMD quintile 5). We would typically expect greater weight relapse with higher deprivation due to socioeconomic factors such as diet and lifestyle [71]. When comparing proportions of weight relapse between lowest and highest 5 IMD deciles, higher deprivation was associated with higher proportions of 10% weight loss at both 5 and 10 years. Differing follow-up rates between patients from different IMD groups may explain this discrepancy. One study found that higher BMI, diabetes, and older age are predictors of increased weight relapse [72], while another study found BMI and diabetes to be significant predictors but not hypertension [73]. Little is known on the effect of deprivation on weight relapse and will need to be investigated further in future studies. The effect of age on weight relapse remains inconclusive as another study reported greater weight relapse in older patients, in contrast to the results of our study [25]. One study classified weight relapse into different grades and found differing patient characteristics in each grade of weight relapse, suggesting that more complex relations based on the severity of weight relapse [41]. Future studies must prioritise frequent and consistent follow-up to better understand the effect of preoperative predictors on weight loss and weight relapse to improve postoperative strategies to maintain long-term weight loss.

### Limitations

Limitations include a retrospective design at a single centre. These findings could be influenced by local practice, patient selection, surgical technique, follow-up protocols and procedure-selection bias. The cohort was predominantly white Caucasian, reducing the accuracy of the effect of ethnicity on weight loss. The study was conducted in a public healthcare setting, reducing its applicability to countries dependent on private healthcare. There was a large disparity in sex among our study cohort with a much larger proportion of female patients. Additionally, there were only 3 separate weight measurements between 2 and 10 years. Finally, our study experienced poor follow-up rates which worsened with increasing length of time. These were 86% at 6 months, 81% at 1 year, 65% at 2 years, 47% at 5 years, and only 29% at 10 years. This introduced a high risk of selection and attrition bias as reasons for non-follow up were not recorded and patients lost to follow up may differ from those retained in analysis. Additionally, this resulted in a small sample size of the long-term cohort, further reducing accuracy of long-term outcomes.

## Conclusions

OAGB has shown comparable weight loss outcomes to RYGB and SG over the short and mid-term. Age, preoperative body mass index, diabetes, operation type and smoking are associated with weight loss up to 2 years after bariatric surgery. Age, hypertension, IMD decile and initial postoperative weight loss are associated with weight relapse after 2 years. Disparity in sex and poor follow-up adherence remain as major challenges that future studies will need to address. Further research on long-term weight loss outcomes after OAGB and the effect of deprivation on weight relapse will need to be conducted.

## Data Availability

No datasets were generated or analysed during the current study.
